# “I had no idea there were psychiatric clinics for children”: A qualitative study of how migrant parents reach Swedish mental health services for their children

**DOI:** 10.1177/13634615241250203

**Published:** 2024-05-23

**Authors:** Ester Gubi, Anna-Clara Hollander, Sofie Bäärnhielm

**Affiliations:** 1 Department of Global Public Health, 27106 Karolinska Institutet, Stockholm, Sweden; 2 Center for Psychiatry Research, Department of Clinical Neuroscience, Karolinska Institutet (KI) and Transcultural Centre, Stockholm Health Care Services, Stockholm, Sweden

**Keywords:** access to services, children, mental health, migration, refugees, qualitative methods

## Abstract

Migrant children have repeatedly been shown to underutilize psychiatric services and to face barriers to care, yet few studies have examined the experience of migrant parents who are successful in their help-seeking efforts for their children's mental health. The aim of this study was to gain a deeper understanding of facilitators and obstacles to reaching care among migrant parents in contact with child psychiatric services. We explored how migrant parents in Stockholm, Sweden, experienced the process of reaching child mental health services. Participants were recruited from out-patient mental health clinics. Ten in-depth interviews were conducted; qualitative analysis of transcripts was undertaken using thematic content analysis. Parents described a desire to reach services but difficulties doing so on their own. We identified a strong dependence on referring agents, such as schools and child health centers, for parents to gain contact. Informants expressed a high degree of trust toward these agents. Contrary to previous studies, stigma was not described as an obstacle to help-seeking but was recognized by informants as a potential barrier to care had they not emigrated. Although participants in our study had differing educational backgrounds and residency times in Sweden, a common experience of reliance on others for reaching services was evident in the data. Our findings highlight the role of referring agents as bridging contacts between different welfare services. Understanding the specific local resources and services that are available to migrant parents, and strengthening these across different sectors, could potentially help reduce barriers to care.

## Introduction

In the past decades, international migration has increased to unprecedented levels and is projected to keep rising (International Organisation for Migration, 2020). Migration to Sweden has been characterized by labor and refugee migration, and until 2015 Sweden had one of Europe's most generous asylum-granting policies (Swedish Migration Agency, 2018). Currently, a fifth of the Swedish population is foreign-born, with the proportion being higher in the larger cities. The most common countries of origin are Syria, Iraq, Finland, Poland, and Iran (Statistics Sweden, 2020).

A growing body of research has consistently shown that refugee children are at increased risk of poor mental health, particularly in terms of depression, anxiety, and post-traumatic stress disorder (Bronstein & Montgomery, 2011; Fazel et al., 2012; Kien et al., 2018). Non-refugee migrant children also face mental health risks due to the psychosocial stress of migration, acculturation, discrimination, and adverse social living conditions (Curtis et al., 2018; Markkula et al., 2018; Oppedal & Idsoe, 2015), and there are indications of poorer mental health among non-refugee migrant children compared to their native peers, though findings have been more inconsistent (Abebe et al., 2014; Curtis et al., 2018; Kim et al., 2020).

Furthermore, research suggests that migrant children and youth reach mental health services at a delay, with a higher risk of in-patient or emergency psychiatric care use (Barghadouch et al., 2016; Montgomery et al., 2020). Moreover, even when migrant children reach care, they may not receive the clinically recommended treatments, compared to native peers with similar diagnoses (Gubi et al., 2022a). Taken together, studies suggest unmet needs, unfavorable service utilization, and inequalities in access to out-patient care and recommended treatments among migrant children. Studying how migrant parents overcome barriers to care could hence deepen our understanding of the challenges they face.

In Sweden, the universal health care system provides free-of-charge health care to all children, irrespective of migration status or residence permit, and on equal terms with all Swedish citizens. Inequalities in service use in Sweden are therefore likely linked to barriers that are not directly of an economic nature. Given that early detection and treatment can help prevent serious and lasting problems (National Institute of Health, 2020), it is worrying that research indicates that migrant children and youth, despite their greater risk of poor mental health, underutilize mental health services. In fact, repeated studies have shown that migrant children use less psychiatric care compared to their majority peers, both in Sweden and other countries (Barghadouch et al., 2016; Berg et al., 2020; Markkula et al., 2018; Montgomery et al., 2020).

There have been efforts to understand barriers to mental health services among young people in general (Gulliver et al., 2010; Radez et al., 2021), and among migrant/minority youth in particular (Satinsky et al., 2019)—revealing that some barriers are common for all. These include concerns about confidentiality, a preference for self-reliance, stigma around mental health problems, lack of knowledge about services, and lack of problem recognition (McCann et al., 2016; Ohtani et al., 2015; Valibhoy et al., 2017; Wang et al., 2019). Nonetheless, certain barriers are more significant for migrants. As such, studies indicate that migrant parents’ perceived lack of adaption to cultural variety among service providers (i.e., perceived lack of culturally competent care based on knowledge of cultural values and practices (Bhui et al., 2007)), differing explanatory models for disease, linguistic obstacles, low trust in the health care system, and limited health literacy act as barriers to care (De Anstiss & Ziaian, 2010; McCann et al., 2016; Napier et al., 2014; Satinsky et al., 2019; Silberholz et al., 2017).

Since mental health service use for minors is influenced by their parents, the role of parents in youth's help-seeking for mental health problems has been investigated. Parental problem recognition has been described as crucial for children and youth's access to mental health services, and disagreement between parents’ and youths’ perception of needs, as well as lack of parental symptom recognition, has been associated with lower use of mental health services among migrant children and adolescents (Verhulp et al., 2013, 2015).

Despite the substantial number of studies investigating potential barriers to care among migrant/minority children, only a small number of studies have examined how migrant children and adolescents reach mental health services (Guzder et al., 2013). For instance, studies have shown that ethnic minority youths in Sweden more often reach child and psychiatric services through referrals from schools, social/legal agencies, and the health care sector compared to Swedish children (Ivert et al., 2011), and teachers have been found to play a significant role in detecting and referring ethnic minority children to child mental health services in Italy (Pedrini et al., 2015). Moreover, a Canadian study investigating how parents reached mental health services for their children's mental health problems found that the majority of parents had started the help-seeking process by turning to physicians and school personnel (Shanley et al., 2008), though this study did not focus specifically on migrant families. Indeed, few studies have explored the experience of migrant parents who have been successful in their efforts to secure mental health care for their children. The aim of this study was thus to explore how migrant parents with children receiving mental health care experienced the process of reaching services.

## Methods

### Setting

Foreign-born parents in contact with mental health services for their children were recruited from out-patient mental health clinics in the Stockholm region. Sweden offers free-of-charge mental health care for all children, irrespective of migration status. In Sweden, psychiatric care for children (0–18 years) is provided at two levels: first-line mental health care, which is organized at the primary care level, for mild to moderate mental health problems, and specialized psychiatric care for serious mental health conditions. Both types of clinic (primary and specialist) can be accessed through self-referral or referrals from other sources (e.g., primary or school-based health care). This applies to all children, regardless of their migration status, though linguistic obstacles may affect the ability of parents/caretakers to make first contact. First contact with services is possible through either telephone or referral. The phone service language is usually restricted to Swedish or English. When contacting a clinic by telephone, the first contact is usually with an automated telephone program that registers the call. Thereafter, a clinic nurse or administrator calls back. For this first contact with the answering machine, it is not possible to request interpreters. Once contact is established, however, interpreters are available for clinical sessions and are free of charge.

The researcher conducting the interviews (EG) initiated collaboration with clinics in neighborhoods with a high proportion of inhabitants born outside of Sweden, by contacting the clinical management and meeting with staff. The clinics were chosen based on available data and local knowledge about the demographic context of the Stockholm region. The municipalities in which these clinics were located had a proportion of foreign-born individuals ranging from 25% to 85%. In total, four clinics participated in the recruitment of informants over a period of three years (2019–2021); three clinics were specialized child and adolescent psychiatric clinics and one clinic was a first-line mental health clinic, located at a primary care center. Two other first-line mental health clinics agreed to participate but were unable to recruit any parents; a few other clinics that were approached declined to participate, without specifying why. Clinics that helped with recruitment of parents participated in the study for varying lengths of time, ranging from six weeks to a full year. Due in part to the fact that migrant children underutilize mental health services and thereby constitute a hard-to-reach group (Berg et al., 2020; Montgomery et al., 2020), recruitment was challenging and required continuous efforts to find clinics to collaborate with.

### Recruitment process and sample

We adopted a purposeful sampling scheme, aimed at finding “information-rich cases” whose participation would yield in-depth understanding of the issues to be investigated (Patton, 2002). Thus, migrant parents who met our predetermined inclusion criteria were asked about participation in the study by their child's clinician. Our inclusion criteria were: i) a maximum of 10 years of residence in Sweden, ii) origin outside of the Nordic countries, and iii) holder of a permanent residence permit. The reason for including only those parents who were holders of permanent permits was to minimize the risks of including participants who, due to their insecure asylum status, could feel uncomfortable declining participation and potentially believe that declining could affect the asylum process or the care of their children. Besides the emotional strain of uncertain asylum outcomes, the asylum-seeking process entails practical obstacles to study participation, such as relocations and potential denial of asylum followed by deportation. We therefore opted to include only permanent residence holders, to reduce ethical and practical risks associated with recruiting participants in the asylum-seeking process.

Moreover, parents whose children were severely ill or suicidal were not approached. Parents were given brief information about the study and asked if they were interested in learning more. Those who agreed were given an information sheet and consented to receiving a telephone call from the researcher conducting the interviews. The information sheet was translated into the most common foreign languages at the different clinics, in dialogue with the clinicians, namely Arabic, English, Somali, Spanish, Tigrinya Turkish, and English. In the subsequent telephone call, detailed information about the study was provided, including thorough information about voluntariness to participate, the right to decline at any point, confidentially, and that declining would not affect the care of their child. Parents who agreed to participate in the interview were scheduled for a session at a time that suited them. An informed consent sheet was discussed with and signed by the participant prior to the interview. The initial telephone call and the interview were conducted with the help of interpreters, at the request of the study participant.

Five parents who agreed to the initial phone call did not participate in an interview. Three of them stated they did not want, or lacked the time, to conduct the interview. One asked for more time to consider participation but did not answer any further calls. The fifth person who declined to participate expressed uncertainty about the purpose of the study. This person's spouse also wished to speak to the researcher, and expressed distrust and skepticism toward authorities in general, and the researcher (EG) in particular.

In total, 10 individual in-depth interviews were carried out with 10 parents. Nine informants were female and one was male. Time of residence in Sweden varied from four months to 10 years; two of the informants had been in Sweden for 10 years, two had been in Sweden for less than one year, two had been in Sweden for four years, and the others had been in Sweden for one, two, three, and five years respectively. In terms of education, participants had varying educational backgrounds, ranging from no formal education to a completed university degree. Living conditions varied from poor housing and employment conditions to stable and economically secure circumstances. The participants’ country of origin included Afghanistan, Brazil (two parents), The Gambia, Iran (two parents), Iraq, Syria, Turkey, and Vietnam.

### Ethics

The study was approved by the Stockholm Regional Ethics Board (permit number: 2017/135-31/5). Due to the COVID-19-outbreak, we switched from conducting face-to-face interviews to telephone interviews during part of the data collection process. We applied and were granted permission for telephone and/or digital interviews in adjustment to the pandemic (permit number: 2020-04180).

### Data collection

Interviews were conducted face to face at a locale of preference to the informant or by telephone. Two informants wished to do the interview at home; the other on-site interviews took place in a room in the first-line mental health clinic. During the COVID-19 pandemic, interviews were conducted by telephone.

Six interviews were conducted in the participant's mother tongue with the help of interpreters, one in Swedish and three in English. The choice of language and use of interpreters was at the participant's discretion. When using interpreters on site, the interpreters were informed about the research project beforehand. Interpreters were engaged from a national firm with certified interpreters in accordance with international standards (ISO 9001:2015, ISO 14001:2015, ISO 27001:2013). Different interpreters were involved in the different interviews. Informants and interpreters were sex matched, apart from in one telephone interview (see the discussion section for an expanded reflection on working with interpreters).

The interpretation of languages was characterized mostly by a passive style, whereby the interpreters interpreted the questions posed by the interviewer and reported back the responses by the informants in the first person. Answers were interpreted in chunks, and sometimes the informant had to be asked to pause for the interpreter to accurately remember what the informant had said. This sometimes required the repetition of answers and an active discussion between interpreter and interviewer. When more detailed answers or clarifications were needed, the interpreters took on a more active role, discussing directly with the informant and interpreting back when informant and interpreter seemed satisfied with having understood each other. The interviewer could then have follow-up questions, which again required the active participation of the interpreter. The main goal was to try to give informants the chance to develop their answers and strive toward an accurate understanding of the questions and answers provided during the interview. We aimed to strengthen the validity of this process by having the interviewer make a great effort to ask for clarifications and to pay attention to possible misunderstandings. Most often, misunderstandings could be identified when the informant expressed uncertainty regarding the questions, or when the interviewer felt unsure of how the answer was related to the questions asked. A recognition and awareness of the greater risks of undetected misunderstandings in the three-person interview situation shaped the interviewer's attention and follow-up questions during the interviews.

We developed a semi-structured interview guide based on the literature reviewed for the study, and the study objectives (see supplementary material 1 for the interview guide). The guide contained key questions that supported the interviewer, while allowing a flexible approach and spontaneity in pursuing the informants’ answers. The main topics covered in the guide included parents’ understanding and description of their child's problems, the informants’ perception of mental illness and help-seeking, their description of coming into contact with mental health services, and their thoughts on whether, and how, the process of gaining access could be made easier. The guide included introducing questions and probing questions, while allowing the interviewer to use follow-up questions where appropriate (Kvale, 2007). The interviews were recorded and transcribed verbatim. They were transcribed in the language of interpretation, i.e., Swedish.

### Reflexivity and trustworthiness

In the present study, the researchers involved had academic backgrounds within medicine, sociology, psychiatry, and epidemiology. Reflections were shared by EG and SB after each interview, to identify ways in which possible pre-understandings, emotional reactions, social interactions, etc. could have influenced the interview and the understanding of the data produced. EG had some previous experience in qualitative interviewing and in conducting clinical interviews with interpreters. SB, who supervised EG, had expertise within qualitative and cultural psychiatry.

The researcher conducting the interviews (EG) was female, with a non-Swedish background, and middle class. Due to the varying backgrounds of the informants, in some of the interviews informants were matched with the interviewer in terms of sex and age, while in others there was matching in social class, and in some in almost no demographic characteristics. Since the interview situation is not a reciprocal interaction between two equal partners, but rather is characterized by a power asymmetry (Kvale, 2007), much care was devoted to trying to ensure that informants understood the purpose of the interview, felt safe, and that any unclarities during the interview were resolved to the best ability of the interviewer.

This last aspect—the effort to resolve uncertainties—was especially important in the interviews conducted with the help of an interpreter. The interpreted interview may pose a threat to validity, especially if an inadequate interpretation is undetected. One way to address this issue is to obtain a second opinion on the interpretation (Ingvarsdotter et al., 2012). For this study, this was achieved by asking a researcher with knowledge of the Arabic language to check the quality of the interpretation in one of the interviews, and this check validated the interpretation of this interview. Furthermore, as described above, much care during the interviews was devoted to ascertaining that the informant had understood the questions and, when uncertainties arose, to clarifying potential misunderstandings. During the interviews, the interviewer regularly checked and summarized what had been conveyed, so as to understand as fully as possible the informant's narrative.

### Data analysis

We applied a thematic analysis, based on Braun and Clarke (2006), to identify and report themes and patterns within the data. The analytical procedure included immersion, data extraction of meaning units, coding of meaning units, and the identifying and reviewing of themes (Braun & Clarke, 2006; Vaismoradi et al., 2013). Our analytical approach was inductive; we strived to maintain an “open” reading of the data which allowed us to arrive at themes that were based on the participants’ own perspectives and not on pre-established theoretical conceptions (Kvale, 2007).

The coding was carried out through an iterative, non-linear process, described here in a stepwise fashion. Part of the immersion process occurred through transcribing the interviews. Transcripts were subsequently read several times for further familiarization with the data. From these readings, data were extracted in the form of sentences that were identified by the main author as key in terms of their ability to answer our research questions or through carrying significant content. This initial data extraction was done after each interview. After this data extraction process, the next step was to gather extracts that were identified as holding a common content into subthemes or themes. When such an initial sub-theme/theme-“draft” had been produced, EG and SB discussed the theme names, contents, and whether the extracted data fit the themes or not. This process was done in an iterative way throughout the analysis process, which was carried out over several months, in parallel with data collection. EG developed preliminary subthemes and themes throughout the data analysis process, which were discussed and revised together with SB on a regular basis, until the interpretation and categorization of the data extracts were agreed on. Thereafter, analysis and themes were discussed with ACH.

An example of extraction of codes and development of a subtheme is provided in Table 1. The data extracts were coded with terms such as “worrying” or “feeling anxious” or “scared.” They were grouped together into the subtheme “expressing feelings of worry and helplessness.” For this subtheme development, much discussion was centered on distinguishing the more “factual” descriptions of the child's difficulties from the emotional reactions that the realization of the child's problems caused. Similar processes of distinguishing commonalities while separating content into distinct themes characterized the entire data analysis process.

**Table 1.** An example of extraction of codes and development of a subtheme.

Data extract	Condensed meaning unit/code	Subtheme	Theme
I’m very worried about it and I feel anxious about her recent behavior. This change, I don’t know about it at all.	Feeling anxious	Expressing feelings of worry and helplessness	Worry and concern
So I took him to the hospital. Because, before, even the school thinks that he has autism. Sometimes when he's running and he's not looking … So I’m scared that there's something wrong with the eyes or …	Scared		
I’m still worried about my son. When the teacher talks about him, I get very worried.	Worrying		

All authors read successive drafts of themes. In the end, again by consensus, five themes were formulated. NVivo 12-software was used for the entirety of the data management and analysis process.

## Findings

Our analysis identified five main themes from the data: i) worry and concern; ii) wanting help and reaching care; iii) trust and stigma; iv) coping with hardship; and v) reflections on accessibility and the care received (see Table 2 for themes, subthemes, and examples of meaning units).

**Table 2.** Themes, subthemes, and example meaning units.

Theme	Subthemes	Example meaning units
**Worry and concern**	Recognizing that the child is unwell Expressing feelings of worry and helplessness	“I’m very concerned. I feel anxious about her behavior” “He became isolated from the other children. He became […] lonely […]” “I don’t know how to help”
**Wanting help and reaching care**	Wanting to get professional help and asking for guidance Obstacles to accessing care Trying to reach services on one's own Reaching services through referral	“I thought we needed a psychologist. So I asked the school for help with a referral” “If she has a problem, I want to know what it is. To help her 100%” “I had no idea there were child and adolescent psychiatric clinics”
**Trust and stigma**	Trusting the health care and educational system Expressing differing familiarity with mental health Rejecting stigma	“If the teacher says it [child's behavior] is not normal, I accept the offer [to get help]” “She [a friend] said the doctors will just give him medication to damage his brain. […] So I stopped talking to people that give me those negative answers” “I wanted to see … just to … maybe understand. So I watched [YouTube] videos [about autism]” “[… ] if you have a psychological problem, you see a psychologist. You are not an ‘idiot’.”
**Coping with hardship**	Coping with loneliness and lack of social support Coping with material difficulties Striving to make things better	“[…] we have no friends, no relatives, who can help us” “We are not used to this kind of economic crisis. In our home country we have jobs, salaries” “We thought that the best thing to do was to go away [… ] go somewhere where we could give the children a secure life”
**Reflections on accessibility and the care received**	Thoughts on how to make access easier Evaluating the care received	“The clinic has been very good. I felt supported the whole time” “Sometimes […] I can see they [the interpreters at the clinic] don’t translate it exactly” “Language is a problem. […] There should be a course about how to seek care […] what to say”

### Theme 1: Worry and concern

The first theme contains data conveying parental worry and concern, including feelings of inability to help one's child on one's own. In this theme, parents described sensing that their child was unwell and expressed feelings of concern and worry connected with this realization. Here, parents described their feelings connected with their child's emotional and behavioral problems. As such, this theme represents the initial stage in the help-seeking process, starting with identification of the child's difficulties and the emotional reactions that this realization entailed. Two subthemes emerged within this theme: *Recognizing that the child is unwell* and *Expressing feelings of worry and helplessness*. The feelings of helplessness that parents expressed were connected to the recognition of the child's problems as well as to a sense of not being able to help the child on one's own.

#### Recognizing that the child is unwell

In describing their child's difficulties, informants gave examples of behavioral and developmental problems, reflecting a variety of mental health problems prevalent among the children. Several parents sensed that their children's behaviors were “not normal” and symptomatic of mental ill-health, and as such, a cause of great concern and worry for them. Below are some of the accounts that parents relayed:

She's acting differently, isolating herself. […] It's strange. […]. She [used to] dance and sing. But now she is so quiet. (Informant 1)

[Because] he's not talking, because some of the kids that he's older than them, they can talk. Like they can “*berätta*” [“tell/narrate” in Swedish]. So, he cannot. (Informant 3)

It was already when he was one and a half years old and we lived in [name of home country] and we noticed that he was very unstill/worried. He could never concentrate on anything … (Informant 6)

#### Expressing feelings of worry and helplessness

Interview participants also recounted a sense of insecurity regarding their capacity to help their children. Along with feelings of concern about their child's well-being, they felt anxious and worried, and described the plight of observing that their children suffered difficulties. Many parents described that the realization that their child may be ill was painful and upsetting, exemplified by these quotes:

As a mother […] you feel bad, because everybody want [sic] their child to be perfect. (Informant 3)

I don’t know how to help him. I feel lost among all my problems. (Informant 4)

Her [the child's] father was also shocked [about the child's changed behavior] and we don’t know what to do. (Informant 1)

### Theme 2: Wanting help and reaching care

The second theme includes data on parents’ desire to obtain help for their children; parents’ descriptions of needing assistance from others to reach services; and the process of reaching care. Here, parents described a desire to get professional help for their children and needing others to access this kind of help. Even though parents felt certain that they needed help, they were unsure of how to obtain mental health care for their children, and thus relied on referrals from others to reach mental health services. This theme is the richest of all the themes in the study and contains four sub-themes: *Wanting to get professional help and asking others for guidance*, *Obstacles to accessing care*, *Trying to reach services on one's own*, and *Reaching services through referral*.

#### Wanting to get professional help and asking others for guidance

For some parents, the desire to get help was grounded in their own observations; for others, staff at school or the primary healthcare center had been the first to alert them of their child's symptoms. Regardless of whether parents had become aware of their child's difficulties on their own or through the attention from school or (non-mental) health care staff, this awareness led to a desire among the parents to obtain professional help for their children. For example, informant 8 had noticed that her child had developmental difficulties and reached out to the primary care nurse to ask for help: “At any rate, I went to them [speaking about the child health care center]. I said that, we, that is, I told them everything I just told, that she didn’t give any eye contact at all” (Informant 8). For informant 3, the child's teachers brought to her attention that her child needed a psychiatric assessment, and this mother expressed that this led her to want to get into contact with services:

Yeah the school you know, they keep insisting that I have to go for [an] assessment because they think that [name of the child] has autism. […] When he told me, I think you have to go for an assessment, I agree, yeah. […] Even though I don’t know I can go for that assessment. But I remember I met that doctor in [name of Stockholm suburb], I told him that I want to go for assessment, cause I’m worried about him …

Informant 1 had a similar experience:

So, in the beginning, it was her [the child's] teacher's suggestion, that we should see a psychologist. […] And then I thought, okay, maybe there is a problem, that she can’t describe. So I said it is better that she sees a psychologist. […]. (Informant 1)

Later in the interview, this parent described that after problems escalated, he “became 100% sure that she needed to see someone” (Informant 1).

#### Obstacles to accessing care

The process of reaching care entailed overcoming uncertainties and practical obstacles. The most salient obstacles to reaching services were unfamiliarity with child and adolescent psychiatric clinics, uncertainty about how to reach a child psychologist/psychiatrist, and language-related difficulties. Several parents described that they realized that their child needed help but were unfamiliar with the organization of the mental health care system in Sweden, and consequently unsure about how to access help. The main obstacles that parents described as hindering them from contacting mental health services were either that they did not know about the existence of these types of clinics, or that they had difficulties reaching services due to linguistic obstacles (not speaking Swedish), as the quotes below illustrate:

I had no idea that BUP [child and adolescent psychiatric clinic] existed. I only knew that there existed psychologists. (Informant 5)

It was difficult for me to understand [the language used in the recorded telephone message]. Because I would call to the primary health center, and then it's always those machines [referring to automatic telephone machines]. (Informant 7)

#### Trying to reach services on one's own

All informants expressed a sense of wanting to get professional help for their children and engaged facilities that they were already familiar with in their help-seeking efforts. The most common places where parents turned were their child's school (teachers or school nurses) or child welfare center (in Swedish, BVC), where they voiced their concerns. Some parents asked directly for referrals to mental health services; others expressed their concerns and asked for help without requesting any specific referral. Moreover, some parents engaged their primary care providers or private networks to make contact with mental health services. As one mother explained, “So I reached out to my friend, and I said, your, does your brother have a psychologist that we could contact?” (Informant 7).

#### Reaching services through referral

Even though all informants had an idea that they wanted to get professional help for their children, some had a clearer notion about the kind of help they wanted than others. Irrespective, however, of whether the informants knew that they wanted psychiatric care, or simply felt that their children needed professional help, they all relied on others to initiate contact with mental health services. Most often this was achieved through referrals from school or primary care professionals, and sometimes through the help of private contacts and friends. Some parents said they believed that their child indeed needed psychological help but did not know how to access such help:

I was at the child health care center, and I told the nurse. After all these [somatic] examinations that they have done. The results were good, but I was not convinced that he was feeling well. He was not doing well. […] So she referred me to “the clinic” [name of the child and adolescent mental health care provider]. (Informant 9)

But I, I thought that we needed a psychologist. And so then we asked the school to send a referral. To ask for a psychologist for children. (Informant 2)

Some informants were less familiar with the concept of child mental health care, but nonetheless wanted medical/professional help to manage their child's difficulties. All informants felt they would have been unable to reach mental health services on their own and described that they had asked for assistance from professionals with whom they were already in contact.

Yes, she [speaking about the child] and her teacher met a doctor at school, and this doctor has asked them, asked us, that we meet this doctor that we have seen […] She first talked to her teacher about this problem, and then they turned, yes, to the school doctor, and the school doctor asked us to go to this doctor, the one we have just seen [at the child and adolescent mental health clinic]. (Informant 6).

And I told her that I don’t know, should I search the internet […] Because I have never visited a psychologist for children. So I asked her what clinics to contact. Then she said that she would send the referral to “the clinic” [name of child and adolescent mental health care provider]. (Informant 8)

Referrals from non-mental health professionals were essential for informants’ contact initiation with mental health services; none accessed these services on their own, through self-referral. No matter if parents had a clear idea that their child needed mental health services, or simply had a perceived need for a professional health care contact, all informants depended on the referring capacity of facilities that they were familiar with, predominantly school and child welfare services:

Because when I search [the internet] a lot comes up, but I don’t know which psychologist is for children, and which is nearby […] So I thought it was best to ask for help. (Informant 8)

They [health care staff in somatic care] explained that, that they are going to have different tests for my son […] before they can provide treatment plan. So they will send us to some department, uh, to uhm, to collect the, to do some evaluation, and collect information. And yeah, so … But I don’t know “the clinic” [name of the child and adolescent mental health clinic], so, uhm, when I received that letter for the appointment, I, and I met the agents in city council, I also showed them that and asked them if this is the place that's for my son from the hospital, but I think that in the letter it also showed that it's the referral from the hospital. (Informant 10)

### Theme 3: Trust and stigma

The third theme contains data on issues of trust and stigma. Here, parents gave accounts of their trust in the medical profession and their rejection of the stigma associated with mental health problems. Three subthemes emerged within this theme: *Trusting the health care and educational system*, *Expressing differing familiarity with mental health*, and *Rejecting stigma*.

#### Trusting the health care and educational system

Except for one mother (informant 4), who thought that her child's problems mainly emanated from inadequate educational support in the school, all parents acknowledged and recognized that their child's difficulties were related to mental health. Informant 4 diverged from the others by regarding her child's problems as not mainly being related to his mental health. She instead thought that the teacher did not adequately understand her child's needs: “I don’t think that a teacher should expect so much from a young child. […] My wish is that they give him [the child] a [another] teacher” (Informant 4). Other parents, who had been alerted by their child's teachers or health care nurses, described that they agreed with the teachers and health professionals regarding their children's needs. Once the school or child-care services recommended further assessment at a mental health clinic, they accepted and appreciated this recommendation. Several informants expressed gratefulness that such help had been proposed by these professionals, as relayed in this example quote: “It was a great suggestion [that the school sent a referral to a child psychiatric clinic]. I am very thankful to the school for showing us M [the name of the psychologist treating the child]” (Informant 2). Moreover, many parents expressed a desire that the child be examined, to obtain optimal help. One mother described that she wanted psychiatric help for her child despite having acquaintances telling her not to see any doctors, as she believed that Swedish doctors were competent and trustworthy. Overall, informants expressed that they trusted and looked forward to the help that the mental health care profession offered, as these informants conveyed:

You have a good chance here, that your child is here, that she will get the correct care. (Informant 8)

This is what I am sure of, that here in Sweden, when you ask for help somewhere, you will get that help. […] I was sure that I will trust them. And that they will do what is necessary. (Informant 9)

Three parents expressed partial disagreement with the mental health professionals’ problem description, yet all accepted the care that was offered. One of these parents believed that the child had mental health issues that required more psychotherapy than he was given, another believed that the child's main issues were connected to language problems in school (as mentioned above), and the third informant questioned the severity of the diagnosis that her child had received. This mother agreed that the child had difficulties, but wanted a new assessment after what she thought was a positive development in the child: “I have asked to see the doctor again, and I have discussed with him that there is a big change, improvement, when it comes to B [name of child]. I ask to be referred. To get a new assessment” (Informant 9). Another mother accepted the diagnosis her child had been given, but at the same time felt that he needed more psychological help and that the diagnosis itself did not fully explain all the child's problems: “He may have ADHD, but … But I don’t think it's only this that's causing all this unhappiness that he claims to feel” (Informant 7). All these informants nonetheless trusted that the care offered would be beneficial to their children and accepted the treatments provided.

#### Expressing differing familiarity with mental health

Even though some parents were not previously familiar with specific mental health disorders, they accepted that their child should be psychiatrically assessed, and believed in the correctness of such assessments. As a mother who had never heard of the psychiatric condition that her child had been diagnosed with explained:

Because I don’t know all these things […] But you know some things, they live in Sweden, they know all these sickness [sic] so they can see the sign. […] Because the doctors, they know, because learned it with their jobs, so. (Informant 3)

Many parents were already familiar with mental health disorders, even prior to the onset of their child's problems. Importantly, however, even those parents who were not previously familiar with mental illness were open to receiving mental health care for their children, accepting the specific diagnosis and treatments offered by the mental health profession.

#### Rejecting stigma

In coming to terms with their child's mental health problems and accepting the provided care, informants rejected any stigma that they nonetheless recognized surrounded mental health issues in society. Informants reported that, in their home countries, mental health was indeed highly stigmatized, and they acknowledged that, to some extent, this issue was salient also in Sweden. For example, one mother reported that she had encountered negative judgment from her acquaintances regarding her child's problems. Nonetheless, parents did not internalize this stigma. Instead, they reported that they spoke about their child's problems with others and that they were not ashamed that their child had mental health needs. This rejection of stigma was often discussed in relation to prejudice in their home countries, and some informants explained that they believed that the lack of education and familiarity with mental health conditions was at the root of stigmatizing practices and notions. The following quotes illustrate the reasoning around stigma from two informants:

[…] one really should stop stigmatizing mental health disorders, because it's an illness like all other illnesses and people don’t want to talk about it. (Informant 7)

[…] first of all one should give information to parents. Because those who move here, perhaps they have still the same prejudice about psychology and such. That's why it's better if […] in school, or Swedish class that they [newly arrived immigrants] attend, inform them that if you have a mental health disorder, you must see a psychologist. You are not an idiot. (Informant 2)

### Theme 4: Coping with hardship

The fourth theme includes data on different kinds of hardship, in the face of which parents still managed to pursue help-seeking for their children's mental health difficulties. In this theme, parents described that they dealt with material and social challenges in terms of poverty, housing insecurity, and lack of social networks, while at the same time striving to make things better for their children. Despite having to deal with substantial social and emotional hardship, parents put much effort into helping their children by securing mental health care for them. This theme emerged during the interviews without having been a central focus of the research questions set out at the start.

Three subthemes were evident within this theme: *Coping with loneliness and lack of social support*, *Coping with material difficulties*, and *Striving to make things better*.

#### Coping with loneliness and lack of social support

For many informants, migration had meant losing the support of friends and family. Even though they were in regular contact with their relatives in their home countries, they nonetheless described a lack of daily support. Many parents described that they had nobody to ask for help in their daily life. Coping with the lack of social networks, some parents described reaching out to parental support groups, the church, or the few friends they did know. For some, the loss of networks was seen as partially responsible for their children's suffering, as well as a hurdle to overcome in terms of seeking care for their children, as exemplified by this mother's account:

We have no family, no relatives here, no acquaintances … Perhaps she [the daughter] misses this bit, the social parts. […] She [the daughter] misses her aunt. […] And this is really important, it's very hard to book a time at the health care center, because we don’t know which way to go … Yes, because we don’t have any friends or family who can help us. (Informant 1)

#### Coping with material difficulties

Many parents expressed a desire to make a better life for their children, while facing many challenges in their new home country. The most prominent issues were insecure housing situations and financial strain, exemplified by the quote below:

The money that I get in child allowance, I use for rent. And we don’t have enough money for food. […] I asked for help, what to do with my children. […] When one moves from one country to another, everything changes. (Informant 4)

Another mother relayed that her children's mental health difficulties were exacerbated by the material circumstances they were in:

It's very hard. We live in a one-bedroom apartment. And both the children have mental … —they are not well. They have psychological problems. Because of this, very small, crowded apartment …. My son needs special treatment, also at home. And he makes things very difficult for his sister. (Informant 1)

#### Striving to make things better

Despite substantial difficulties, parents expressed a desire to help their children and try to make a good life for them, both in terms of their social and material situation, as well as regarding their mental wellbeing. This desire was expressed as their rationale for migrating, and in their efforts to secure help and treatment for their children:

We thought that the best thing to do was to go away […] Go somewhere where we could give the children a secure life. (Informant 6)

We have some options, migration options. To US, and to other countries. But we decided to move to Sweden because of this. [Because] I hear about the best caring system, and services, here in Sweden. (Informant 10)

### Theme 5: Reflections on accessibility and the care received

The final theme contains information on parents’ thoughts about accessing care, their reflections on the process of reaching mental health services, and their evaluation of the care received. For all informants, the challenge had been to gain initial contact with services; once this was established, the care received had been satisfying to most parents. Two subthemes were developed in this theme: *Thoughts on how to make access easier* and *Evaluating the care received*.

#### Thoughts on how to make access easier

As reported above, the most prominent difficulties with reaching services had been knowing which services to contact and being able to do so in a foreign language. Suggestions regarding how services could be easier to reach pertained chiefly to language. Several informants described that reaching services by telephone when not knowing Swedish should be made easier and that the automatic telephone machines (that are standard in most out-patient clinics in Stockholm) should be adapted to function in different languages. Others suggested that information about specific child mental health clinics should be made more comprehensible and detectable online. One informant thought that the clinics should become more visible in the community. Lastly, education and information targeted at newly arrived immigrants about mental health services for children was suggested to improve access to and reachability of services, especially in the form of a website with relevant and easily accessible information. As one mother put it: “[…] There should be a centralized website, or link, that can provide full guidance for the immigrants… […] I think that there are different websites to contact for different purposes. […] They should consolidate” (Informant 10).

##### Evaluating the care received

Most parents expressed satisfaction with the care their children had received and described that once they had established contact with services, there had been little difficulty communicating or getting the help they wanted. Many parents described that they managed to communicate with providers without complete mastery of the Swedish language, and several used interpreters in the sessions without encountering difficulties. Most parents said that they had received the help they had hoped to obtain and were content with the process, once the initial difficulty of gaining access had been overcome: “I thought that the child and adolescent clinic was very good. I felt supported the whole time. […] I could call and ask my questions, and always got answers” (Informant 8). However, two parents described negative encounters during visits to the clinics. One informant recounted that she had had negative experiences with interpreters on several occasions. She had declined using interpreters after sessions with interpreters who had stated their own, often negative views, rather than interpreting what was being said. This informant was nonetheless satisfied with the care that her child had received. She had asked to speak English with the providers after experiencing bad sessions with inadequate interpreters—and felt satisfied with this strategy. The other informant who expressed having had bad experiences with providers told of an encounter with a psychiatrist whom she felt had behaved rudely and negligently toward her. She expressed that she felt discomfort when speaking to this doctor, but that she had had no problems with the other clinicians with whom she had also been in contact, and whom she trusted and appreciated: “I was uncomfortable meeting this doctor. But […] when I met the others [names two other staff at the clinic], it was very easy and very nice to meet them” (Informant 9).

## Discussion

In this qualitative interview study, we explored migrant parents’ experiences of reaching mental health services for their children. We found that parents experienced worry and concern for their children, wished to get professional help but felt uncertain how to obtain it, and relied on referring agents to gain access. Moreover, parents expressed trust toward professionals with whom they were already in contact, primarily teachers and child health care personnel, and toward the mental health care system in general, despite differing levels of prior knowledge regarding mental health problems. The barriers to care were thus of access; once services had been reached, the treatments were accepted and often thankfully received.

A key finding in the present study is that referring agents were crucial for contact initiation with mental health services for children and their parents. Notably, many parents described that either they had turned to their child's school to ask for help or the school had suggested a referral, which parents readily accepted. Previous research has shown that schools could indeed play an important role in enhancing access to mental health services. As such, a review of barriers to care among vulnerable groups identified the potential of school-based interventions in making mental health services available to a broad population (Silberholz et al., 2017), while a study of pathways to child and mental health services in Italy showed that immigrant youth were more often referred by teachers compared to their majority peers (Pedrini et al., 2015). This study also concluded, much in line with our findings, that schools were central for referring minority ethnic youth with mental health problems to services, given that their parents may have limited information about the workings of the mental health service system (Pedrini et al., 2015). Our results correspond to these, and we thereby strengthen the body of literature that stresses the importance of schools (and other local institutions) in enhancing access to mental health services for families who may be less familiar with the functions of the health care system.

Informants talked about unfamiliarity with mental health clinics and how to access these, while some parents also told that they had little previous knowledge about mental health. These results are in line with previous findings showing that barriers to mental health help-seeking among refugees include a lack of understanding of how to access services (Byrow et al., 2020); that limited knowledge of the mental health care system impedes migrant families from understanding how to seek care for their children (Place et al., 2021a); and that low parental mental health literacy acts as a barrier to help-seeking among migrant youths and their families (McCann et al., 2016). In fact, our findings correspond clearly to those found in a recent review in which participants expressed that, even with a basic awareness of services, parents may not know where to start the help-seeking process (Place et al., 2021a). Consistent with this finding, several informants in the present study reported that, despite a general knowledge of the existence of mental health services for children, this awareness itself was not enough to generate access. Importantly, thus, our findings highlight the potential benefit of strengthening the bridging capacity of local institutions that are known and available to migrant parents; using these, parents were able to overcome barriers to mental health services.

Notably, our results diverge from previous studies regarding stigma. A large body of literature has documented stigma as a significant barrier to care among migrant as well as among non-migrant youths and their families (Bradby et al., 2007; Byrow et al., 2020; Place et al., 2021a; Posselt et al., 2017; Radez et al., 2021; Valibhoy et al., 2017). The informants in our study were aware of stigma as a societal problem but did not report it as a barrier to help-seeking. Furthermore, they acknowledged that in their home countries stigma would have been a more serious obstacle to their help-seeking—in congruence with previous findings showing that migrant parents are more open to seeking mental health care in their countries of resettlement than in their countries of origin (AlAzzam & Daack-Hirsch, 2015). Important to note is that the informants in the present study constitute a selected group of parents who wanted contact with mental health services. As such, they were likely to be more prone to resisting stigma. Consequently, had we been able to interview parents of children with mental health difficulties who despite their children's issues deferred help-seeking, our findings on stigma may have been different.

While the selection of the sample is crucial to bear in mind, there are indications that stigma is not always the main barrier to care among migrant or non-migrant populations (Olsson et al., 2020). In a study of unmet needs and help-seeking for mental problems among adults in Sweden, several factors were found to be more important barriers to care than stigma. Low perceived needs, low trust in the effectiveness of mental health care, and lack of knowledge of where to seek care were the most prominent barriers to help-seeking. Moreover, stigma was not reported more frequently as a barrier to care among those with non-Nordic origins compared to those with Nordic countries of birth (Olsson et al., 2020). Studies also show that with increased parental time of residence, the likelihood of receiving care among migrant children increases (Finno-Velasquez et al., 2016), indicating the complex interconnections of migration experiences, time of residence, and help-seeking behaviors.

Since parents in the present study reported that they had not known how to initiate contact with services, and a lack of knowledge of services has been shown to impede help-seeking (Byrow et al., 2020; Olsson et al., 2020; Place et al., 2021a), efforts to increase accessibility of mental health services for migrant families seem warranted. Examples of such endeavors are the health communicators available within the Stockholm region at the Transcultural Center, who offer information to newly arrived immigrants about the Swedish health care system, and an on-going research project aiming to lower barriers to primary mental health services for migrant children (Place et al., 2021b)—but overall, the lack of information campaigns is notable. The dependence on referring agents described by the informants in our study may indeed reflect the need for systematic outreach and educational efforts to improve parental ability to access services for their children.

### Strengths and limitations

As described above, participants in this study shared a sense of trust toward professionals in schools and primary care centers, with whom they were already familiar. Learning about their path into care can illuminate factors that could facilitate the process for other migrant parents in similar contexts. However, from this study, we cannot learn about the barriers experienced by migrant parents who do not reach services, even if their children need help. This is clearly a limitation in our study, and a subject matter that should be explored in continued research efforts (for studies that have investigated this, see for example Bradby et al., 2007).

Moreover, the sample consisted of migrant parents with varying demographic and migration backgrounds. Given that previous research has shown that country of origin and time of residence in the home country affects utilization of mental health services (Berg et al., 2020; Ivert et al., 2013), the constitution of the present sample may have affected the accounts that we elicited. A larger number of informants, or a higher degree of homogeneity among them, may have yielded different results. The limitations regarding transferability of our findings should thus be considered with the variation of the present sample, as well as its smaller size, in mind.

### Interviewing face to face and by telephone

The COVID-19 pandemic made face-to-face interviews impossible during part of this study's data collection period, and we switched to conducting interviews at distance. Telephone interviews imply no possibility for non-verbal communication (besides silences), and consequently cues and meanings that can be observed and analyzed in the face-to-face setting are lost. Although this represents a limitation, the decision to continue the work by telephone interviews meant that informants could nonetheless share their accounts.

### On the role of the interpreter

Ensuring a correct understanding of what the informants told, such that the findings justly reflect what informants conveyed, involved challenges due to the presence of interpreters. While undertaking interviews with interpreters is an important way of including non-native speakers in research (Plumridge et al., 2012), there may be certain “costs” in terms of loss of control, spontaneity, small-talk, and rapport-building (Aranguri et al., 2006; Plumridge et al., 2012). In addition, interpretation may be a threat to validity, for instance when cultural differences, disparate “coherence systems,” and an inadequate interpretation cannot be detected or resolved (Ingvarsdotter et al., 2012; Kapborg & Berterö, 2002). For this study, we carried out an informal evaluation of one of the interviews, whereby a native Arabic speaker in the research group (not involved in the study) was asked to listen to parts of an interview with an Arabic interpreter and verified the validity of the interpretation.

Some of the above-mentioned challenges were nonetheless evident in the interviews. Thus, in one interview where the informant had asked for an interpreter in Persian, the interpreter had difficulties understanding some of the informant's speech. The informant explained that her mother tongue was Dari but that she preferred to speak Persian. The interpreter stated that she could interpret both Dari and Persian (the two being very closely related), yet found the interpretation difficult, perhaps due to unusual nuances in dialect. During this interview, the three of us (EG, the informant, and the interpreter) addressed the problem openly and took time for clarifications, but it is possible that some loss of meaning occurred. In another interview, the informant, who had some knowledge of the Swedish language, sometimes corrected the interpreter directly and thereby “exercised the power dynamic to his favor,” illuminating the complex dynamics of interaction that occur in the interpreted interview situation (Edwards, 1998).

Lastly, in all the interviews, much care was devoted to checking that the informant had understood the questions and, when uncertainties arose, to trying to clarify potential misunderstandings. Regularly during the interview, the interviewer checked back with the informant that she had understood correctly what had been said, by summarizing and giving a brief interpretation of what had been conveyed. This “checking” was an attempt to validate the interviewer's understanding of the informant's narratives.

## Conclusion and implications

This study provides an understanding of experienced barriers and access-enhancing factors among migrant parents in contact with mental health services for their children. It yields insight into how parents negotiate issues of stigma, and handle help-seeking in a context of unfamiliarity and linguistic obstacles. Although the results of this qualitative study cannot be generalized, exploring service access through qualitative enquiry can elucidate some of the barriers to care that have been indicated in epidemiological studies—which have consistently shown underutilization of mental health services among migrant youth (Berg et al., 2020; Gubi et al., 2022b; Montgomery et al., 2020)—and potentially guide future research into the underlying mechanisms behind the observed disparities.

For the parents participating in this study, referring agents were central in providing access to mental health services. Moreover, the trust toward institutions with which parents were already familiar (e.g., schools and child welfare centers) played a key role in parents’ help-seeking processes. Illuminating the course by which informants reached services sheds light on the importance of cooperation between different welfare services. Although welfare systems look different in different settings, an understanding of the local services with which migrant parents are in contact, and a possible strengthening of such resources, could be a potential focus for policies aimed at reducing barriers to care. Future research on programs that facilitate contact between different services/institutions and that build on trust could potentially enhance access to care among groups who commonly face barriers. Community-based interventions with the aim of increasing health literacy and lowering barriers should be further explored and developed (for a current example, see Place et al., 2021b).

Given that informants for the present study were recruited from mental health clinics and as such constituted a selected group of parents, it is likely that a wider sample of informants—including parents from other settings—would provide more information about barriers to care. Future research would benefit from studying migrant parents whose children may need help but who have not reached services. Accounts from such parents could advance our understanding of factors that impede contact initiation and ultimately guide policy to make mental health services more equitably available and accessible for all.

## Supplemental Material

sj-docx-1-tps-10.1177_13634615241250203 - Supplemental material for “I had no idea there were psychiatric clinics for children”: A qualitative study of how migrant parents reach Swedish mental health services for their children Supplemental material, sj-docx-1-tps-10.1177_13634615241250203 for “I had no idea there were psychiatric clinics for children”: A qualitative study of how migrant parents reach Swedish mental health services for their children by Ester Gubi, Anna-Clara Hollander and Sofie Bäärnhielm in Transcultural Psychiatry
