# Supplementary material for: “I had no idea there were psychiatric clinics for children”: A qualitative study of how migrant parents reach Swedish mental health services for their children
Source: Transcult Psychiatry. 2024 May 23;61(6):842–57. doi: 10.1177/13634615241250203 (PMC11664882; doi:10.1177/13634615241250203)
Supplement: sj-docx-1-tps-10.1177_13634615241250203 - Supplemental material for “I had no idea there were psychiatric clinics for children”: A qualitative study of how migrant parents reach Swedish mental health services for their children [file sj-docx-1-tps-10.1177_13634615241250203.docx]

**Interview guide**

1. To start with, I’d like to ask some questions about you.

How old are you?

Where were you born?

When did you leave your home country and when did you arrive in Sweden?

Do you work? (What do you do?)

(Education?)

What is your mother tongue?

What language do you speak with your children?

What other languages do you speak?

What is your family situation?

How many children do you have

How old are they?

What does your living situation look like?

Do you have stable housing?

How do you live with?

Does religion play any role in your life?

Now I would like to ask about your child.

1. How is your child?

Could you tell me how it all started?
 How did you notice that your child was unwell?

Could you describe the difficulties your child had/has?

I’d like to ask a bit more about your child’s difficulties.

1. What have you found out about your child’s problems?

What do you call your child’s problems?

What have you been told from the health care staff?

What does ”name of problem” mean to you?

(N.B. use informant’s own description)

How did you experience your child’s difficulties?

Do you think the child has a problem?

How do other people react (family, friends, school, others)?

1. What do you think are the causes of your child’s difficulties?

Is there anything that happened that may have contributed to your child’s problems?

Do you think there may be other factors that mattered?

What do you think other people think about your child’s problem?

(People in school, friends and acquaintances, health care professionals)?

1. Can you tell me how you came into contact with the clinic?

What did you do to gain contact?

Did you contact them directly?

Did you get recommendations/tips/advice? From whom?

Referrals?

Did anyone outside the family notice that the child was unwell?

1. Did you/how did you try to help your child before coming to the clinic?

What did you do?

Whom did you talk to?
Was there anyone that you did *not* want to talk to? Why not?

What do you think other people think about your getting help at this clinic?

1. Where did you first turn when you wanted to get help?

How did you go about to seek information?

Whom did you talk to?

1. Did you seek help from anywhere else? Can you tell me about that?
2. What would you have done in your home country?
3. How would you describe the process of reaching the clinic?

Was it hard? Easy?

Was language an issue?

Were there other difficulties? Things that were good?

1. What has it been like to talk the staff?

Do you feel you have been heard?

Do you feel that you have been able to ask questions?
Get answers?

1. Is there anything that would have made it easier to gain contact with the clinic/the mental health care-system?

Is there any help/support you wish would have been available?

(In schools, child health care centers, anywhere else?)

Did you use the internet? How was that?

1. Do you feel that you have received the help you needed?
2. What would you tell a friend who asked for advice regarding their child (with similar difficulties as your own)?
3. Is there anything else you would like to mention?
4. What was it like for you to talk about all of this with me?
